# Attitudes towards a Compassion Focus Therapy Group for Psychosis: A Survey of Service Users and Clinicians

**DOI:** 10.1192/j.eurpsy.2023.2294

**Published:** 2023-07-19

**Authors:** N. Slingerland, C. Twomey

**Affiliations:** Psychology, St Patricks Mental Health Services, Ireland, Dublin, Ireland

## Abstract

**Introduction:**

The Living Through Psychosis (LTP) programme at St Patrick’s Mental Health Services, Ireland (SPMHS) is heavily informed by the Compassion Focus Therapy (CFT) for Psychosis model. LTP offers an opportunity for service users to develop compassion skills to cope with emotional and psychological challenges relating to living with psychosis; and to develop their capacity to for a mindful, non-judgemental and compassionate awareness of distressing thoughts and images.

**Objectives:**

This (ongoing) online survey explores both service user and clinician attitudes towards the CFT-informed LTP group. We also aim to identify any potential concerns that might demotivate referrals to LTP and similar programmes, and to explore what are judged to be its benefits. The study also provides an opportunity to develop and improve the LTP programme to best fulfil service users’ needs.

**Methods:**

The online survey is concise and responses are anonymous. Clinicians and service users complete similar-but-separate sets of questions that are adapted for relevancy and wording. The survey mainly consists of Likert Scale questions in relation to potential participation in, or referral to, LTP (after a visually-aided description of LTP is provided online within the survey). Using convenience sampling, the survey has been distributed among clinicians and service users through email and Internet advertisements within SPMHS and psychosis organisations such as Psychosis Ireland. Descriptive analysis is used for quantitative questions, while thematic analysis covers qualitative questions.

**Results:**

Data collection is currently ongoing and will finish in December 2022. Preliminary results will be presented at the conference.

**Conclusions:**

Conclusions will be derived from the results. It is anticipated that the findings will be helpful in further developing the LTP programme and similar CFT programmes for psychosis.

**Disclosure of Interest:**

None Declared

